# Bis[2-phenyl-1-(phenyl­iminio)isoindo­line] di-μ-chlorido-bis­[dichloridopalladate(II)] benzene disolvate

**DOI:** 10.1107/S1600536808017005

**Published:** 2008-06-13

**Authors:** Jackson M. Chitanda, J. Wilson Quail, Stephen R. Foley

**Affiliations:** a Department of Chemistry, University of Saskatchewan, 110 Science Place, Saskatoon, Saskatchewan, Canada S7N 5C9; b Saskatchewan Structural Sciences Centre, University of Saskatchewan, 110 Science Place, Saskatoon, Saskatchewan, Canada S7N 5C9

## Abstract

In the title compound, (C_20_H_17_N_2_)_2_[Pd_2_Cl_6_]·2C_6_H_6_, the dichloride-bridged [Pd_2_Cl_6_]^2−^ anion lies across an inversion center with each Pd^II^ ion in a slightly distorted square-planar environment. In the crystal structure, two cations and an anion are connected *via* N—H⋯Cl hydrogen bonds between the NH groups of the iminioisoindoline cations and terminal Cl atoms of a hexa­chloridodipalladate(II) anion. The Pd—Cl distance of the terminal chloride engaged in hydrogen bonding is slightly longer than the Pd—Cl distance of the adjacent terminal chloride which is not involved in hydrogen bonding.

## Related literature

For related literature, see: Bartczak *et al.* (2001 ▶); Chitanda *et al*. (2008 ▶); Fábry *et al.* (2004 ▶); Lassahn *et al.* (2003 ▶); Ojwach *et al.* (2007 ▶); Schupp *et al.* (2001 ▶); Yang *et al.* (2008 ▶).
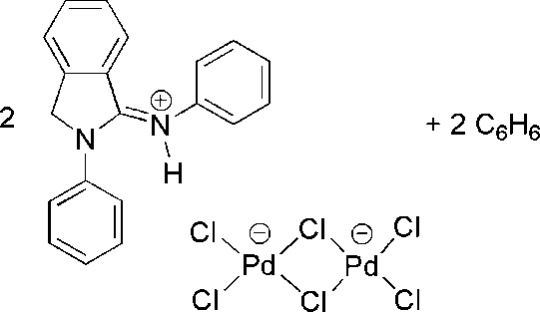


## Experimental

### 

#### Crystal data

(C_20_H_17_N_2_)_2_[Pd_2_Cl_6_]·2C_6_H_6_
*M* *_r_* = 1152.46
Triclinic, 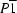

*a* = 9.5457 (3) Å
*b* = 9.9754 (3) Å
*c* = 14.8002 (5) Å
α = 74.270 (2)°
β = 80.615 (2)°
γ = 63.228 (2)°
*V* = 1209.74 (7) Å^3^
*Z* = 1
Mo *K*α radiation
μ = 1.11 mm^−1^
*T* = 173 (2) K
0.22 × 0.18 × 0.05 mm

#### Data collection

Bruker–Nonius KappaCCD diffractometer
Absorption correction: ψ scan (*SHELXTL*; Sheldrick, 2008 ▶) *T*_min_ = 0.791, *T*_max_ = 0.946
18458 measured reflections
6458 independent reflections
5322 reflections with *I* > 2σ(*I*)
*R*_int_ = 0.038

#### Refinement

*R*[*F* ^2^ > 2σ(*F* ^2^)] = 0.039
*wR*(*F* ^2^) = 0.094
*S* = 1.04
6458 reflections
290 parameters
H-atom parameters constrained
Δρ_max_ = 0.82 e Å^−3^
Δρ_min_ = −0.94 e Å^−3^

### 

Data collection: *COLLECT* (Nonius, 1998 ▶); cell refinement: *SCALEPACK* (Otwinowski & Minor, 1997 ▶); data reduction: *DENZO* (Otwinowski & Minor 1997 ▶) and *SCALEPACK*; program(s) used to solve structure: *SIR97* (Altomare *et al*., 1999 ▶); program(s) used to refine structure: *SHELXL97* (Sheldrick, 2008 ▶); molecular graphics: *ORTEP-3 for Windows* (Farrugia, 1997 ▶); software used to prepare material for publication: *SHELXL97*.

## Supplementary Material

Crystal structure: contains datablocks I, global. DOI: 10.1107/S1600536808017005/lh2628sup1.cif

Structure factors: contains datablocks I. DOI: 10.1107/S1600536808017005/lh2628Isup2.hkl

Additional supplementary materials:  crystallographic information; 3D view; checkCIF report

## Figures and Tables

**Table:** 

Pd1—Cl2	2.2635 (7)
Pd1—Cl1	2.2929 (7)
Pd1—Cl3^i^	2.3292 (7)
Pd1—Cl3	2.3374 (7)

**Table:** 

Cl2—Pd1—Cl1	91.32 (3)
Cl2—Pd1—Cl3^i^	91.00 (3)
Cl1—Pd1—Cl3^i^	177.33 (3)
Cl2—Pd1—Cl3	176.86 (3)
Cl1—Pd1—Cl3	91.47 (3)
Cl3^i^—Pd1—Cl3	86.25 (3)

Symmetry code: (i) 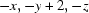.

**Table 2:** Hydrogen-bond geometry (Å, °)

*D*—H⋯*A*	*D*—H	H⋯*A*	*D*⋯*A*	*D*—H⋯*A*
N2—H2⋯Cl1	0.88	2.37	3.242 (2)	171

Symmetry codes: .

**Fig. 1.**
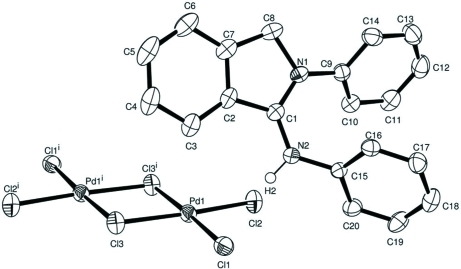
Molecular structure of the title compound with thermal ellipsoids at the 50% probability level. H atoms not participating in H-bonding are omitted for clarity. Only the symmetry unique cation is shown [symmetry code: (i) -x, -y+2, z].

**Fig. 2.**
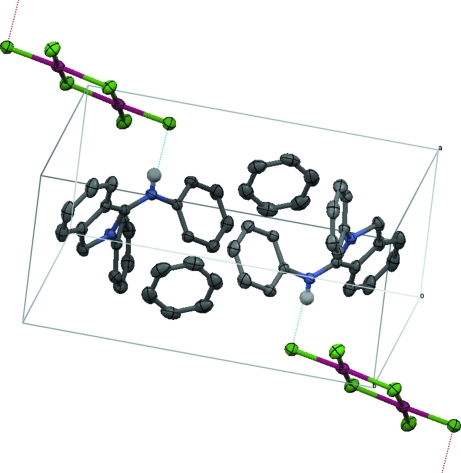
Packing of the title compound with hydrogen bonds shown with dashed lines. H atoms not participating in H-bonding are omitted for clarity.

## supplementary crystallographic information

### Comment

As part of the ongoing research in our laboratory directed at the synthesis of substituted palladacycles incorporating iminoisoindolines (Chitanda *et al.*, 2008), the title compound, I, was obtained by reaction of 1-phenylimino-2-phenylisoindoline with dichloropalladium(II) in the presence of HCl. The bis-iminoisoindolinium hexachlorodipalladate complex crystallizes with two molecules of benzene in the unit cell of the triclinic space group P1. The crystal structure of I is stabilized by a system of intermolecular hydrogen bonds between the imine NH atoms of the iminoisoindolinium cation and the termininal chloride atoms in the hexachlorodipalladate(II) anion. The Pd_2_Cl_6_^2-^ anion lies across an inversion center and has the expected planar dichloro-bridged structure with the Pd—Cl distance of the terminal chloride engaged in hydrogen bonding being slightly longer at 2.2929 (7)Å than the Pd—Cl distance of the adjacent terminal chloride at 2.2635 (7)Å which does not show any H-bonding. In previously reported structures incorporating a Pd_2_Cl_6_^2-^ anion, the anion most often lies across an inversion center (Bartczak *et al.*, 2001; Fábry *et al.*, 2004; Lassahn *et al.*, 2003; Ojwach *et al.*, 2007; Schupp *et al.*, 2001; Yang *et al.*, 2008). The molecular structure and packing of the title compound is shown in Figs. 1 and 2.

### Experimental

The title compound was synthesized by reaction of 1-phenylimino-2- phenylisoindoline with dichloropalladium(II) in the presence of HCl in dichloromethane. Single crystals were obtained by slow evaporation from a benzene solution at ambient temperature.

### Refinement

H atoms were placed in calculated positions with *U_iso_* constrained to be 1.2 times *U_eq_* of the carrier atom for all hydrogen atoms.

### Crystal data

**Table:**

(C_20_H_17_N_2_)_2_[Pd_2_Cl_6_]·2C_6_H_6_	*Z* = 1
*M**_r_* = 1152.46	*F*_000_ = 580
Triclinic, *P*1	*D*_x_ = 1.582 Mg m^−^^3^
Hall symbol: -P 1	Mo *K*α radiation λ = 0.71073 Å
*a* = 9.5457 (3) Å	Cell parameters from 5512 reflections
*b* = 9.9754 (3) Å	θ = 1.0–29.1º
*c* = 14.8002 (5) Å	µ = 1.12 mm^−^^1^
α = 74.270 (2)º	*T* = 173 (2) K
β = 80.615 (2)º	Plate, orange
γ = 63.228 (2)º	0.22 × 0.18 × 0.05 mm
*V* = 1209.74 (7) Å^3^	

### Data collection

**Table:**

Bruker–Nonius KappaCCD diffractometer	6458 independent reflections
Radiation source: fine-focus sealed tube	5322 reflections with *I* > 2σ(*I*)
Monochromator: horizonally mounted graphite crystal	*R*_int_ = 0.038
Detector resolution: 9 pixels mm^-1^	θ_max_ = 29.1º
*T* = 173(2) K	θ_min_ = 2.9º
φ scans and ω scans with κ offsets	*h* = −13→11
Absorption correction: ψ scan(SHELXTL; Sheldrick, 2008)	*k* = −13→13
*T*_min_ = 0.791, *T*_max_ = 0.946	*l* = −20→20
18458 measured reflections	

### Refinement

**Table:**

Refinement on *F*^2^	Hydrogen site location: inferred from neighbouring sites
Least-squares matrix: full	H-atom parameters constrained
*R*[*F*^2^ > 2σ(*F*^2^)] = 0.039	*w* = 1/[σ^2^(*F*_o_^2^) + (0.0328*P*)^2^ + 1.4367*P*] where *P* = (*F*_o_^2^ + 2*F*_c_^2^)/3
*wR*(*F*^2^) = 0.094	(Δ/σ)_max_ < 0.001
*S* = 1.04	Δρ_max_ = 0.82 e Å^−^^3^
6458 reflections	Δρ_min_ = −0.94 e Å^−^^3^
290 parameters	Extinction correction: SHELXL97 (Sheldrick, 2008), Fc^*^=kFc[1+0.001xFc^2^λ^3^/sin(2θ)]^-1/4^
Primary atom site location: structure-invariant direct methods	Extinction coefficient: 0.0061 (7)
Secondary atom site location: difference Fourier map	

### Special details

**Table:**

Geometry. All e.s.d.'s (except the e.s.d. in the dihedral angle between two l.s. planes) are estimated using the full covariance matrix. The cell e.s.d.'s are taken into account individually in the estimation of e.s.d.'s in distances, angles and torsion angles; correlations between e.s.d.'s in cell parameters are only used when they are defined by crystal symmetry. An approximate (isotropic) treatment of cell e.s.d.'s is used for estimating e.s.d.'s involving l.s. planes.
Refinement. Refinement of *F*^2^ against ALL reflections. The weighted *R*-factor *wR* and goodness of fit *S* are based on *F*^2^, conventional *R*-factors *R* are based on *F*, with *F* set to zero for negative *F*^2^. The threshold expression of *F*^2^ > σ(*F*^2^) is used only for calculating *R*-factors(gt) *etc*. and is not relevant to the choice of reflections for refinement. *R*-factors based on *F*^2^ are statistically about twice as large as those based on *F*, and *R*- factors based on ALL data will be even larger.

### Fractional atomic coordinates and isotropic or equivalent isotropic displacement parameters (Å^2^)

**Table:**

	*x*	*y*	*z*	*U*_iso_*/*U*_eq_	
Pd1	0.00603 (2)	0.87175 (2)	0.103892 (15)	0.02846 (8)	
Cl1	0.05538 (8)	0.85027 (8)	0.25493 (5)	0.03459 (15)	
Cl2	−0.03111 (9)	0.65391 (8)	0.14749 (5)	0.03895 (17)	
Cl3	0.04870 (8)	1.09399 (8)	0.05063 (5)	0.03515 (15)	
N1	0.5133 (2)	0.3157 (3)	0.18312 (15)	0.0261 (4)	
N2	0.3165 (2)	0.4983 (2)	0.26541 (15)	0.0263 (4)	
H2	0.2504	0.5964	0.2559	0.032*	
C1	0.4296 (3)	0.4577 (3)	0.19937 (17)	0.0243 (5)	
C2	0.4887 (3)	0.5622 (3)	0.13560 (18)	0.0284 (5)	
C3	0.4427 (4)	0.7181 (4)	0.1276 (2)	0.0355 (6)	
H3	0.3529	0.7768	0.1619	0.043*	
C4	0.5329 (4)	0.7844 (4)	0.0678 (2)	0.0435 (7)	
H4	0.5049	0.8908	0.0608	0.052*	
C5	0.6646 (4)	0.6971 (4)	0.0176 (2)	0.0471 (8)	
H5	0.7254	0.7453	−0.0223	0.057*	
C6	0.7087 (4)	0.5426 (4)	0.0246 (2)	0.0418 (7)	
H6	0.7979	0.4843	−0.0103	0.050*	
C7	0.6181 (3)	0.4753 (3)	0.08448 (19)	0.0317 (6)	
C8	0.6411 (3)	0.3118 (3)	0.1107 (2)	0.0332 (6)	
H8A	0.6299	0.2787	0.0560	0.040*	
H8B	0.7455	0.2421	0.1366	0.040*	
C9	0.4809 (3)	0.1840 (3)	0.21669 (18)	0.0267 (5)	
C10	0.3283 (3)	0.1986 (3)	0.22435 (19)	0.0300 (5)	
H10	0.2433	0.2972	0.2072	0.036*	
C11	0.3010 (4)	0.0682 (4)	0.2573 (2)	0.0381 (6)	
H11	0.1967	0.0770	0.2640	0.046*	
C12	0.4264 (4)	−0.0752 (4)	0.2804 (2)	0.0428 (7)	
H12	0.4072	−0.1643	0.3037	0.051*	
C13	0.5781 (4)	−0.0902 (3)	0.2699 (2)	0.0425 (7)	
H13	0.6633	−0.1894	0.2840	0.051*	
C14	0.6063 (3)	0.0394 (3)	0.2387 (2)	0.0352 (6)	
H14	0.7109	0.0299	0.2324	0.042*	
C15	0.2902 (3)	0.4009 (3)	0.34982 (17)	0.0233 (5)	
C16	0.4162 (3)	0.2904 (3)	0.40237 (18)	0.0269 (5)	
H16	0.5201	0.2780	0.3821	0.032*	
C17	0.3886 (3)	0.1979 (3)	0.4851 (2)	0.0338 (6)	
H17	0.4743	0.1212	0.5216	0.041*	
C18	0.2376 (4)	0.2166 (4)	0.5149 (2)	0.0379 (6)	
H18	0.2198	0.1519	0.5713	0.045*	
C19	0.1122 (3)	0.3291 (4)	0.4628 (2)	0.0381 (7)	
H19	0.0083	0.3422	0.4839	0.046*	
C20	0.1372 (3)	0.4233 (3)	0.3796 (2)	0.0314 (6)	
H20	0.0512	0.5014	0.3439	0.038*	
C21	0.6984 (4)	0.4602 (4)	0.4106 (3)	0.0488 (8)	
H21	0.6432	0.5641	0.4167	0.059*	
C22	0.7139 (4)	0.3430 (4)	0.4878 (3)	0.0457 (8)	
H22	0.6699	0.3660	0.5472	0.055*	
C23	0.7927 (4)	0.1921 (4)	0.4801 (3)	0.0455 (8)	
H23	0.8031	0.1110	0.5338	0.055*	
C24	0.8565 (4)	0.1594 (4)	0.3940 (3)	0.0480 (8)	
H24	0.9110	0.0553	0.3883	0.058*	
C25	0.8418 (4)	0.2765 (5)	0.3164 (3)	0.0532 (9)	
H25	0.8862	0.2535	0.2571	0.064*	
C26	0.7618 (4)	0.4292 (5)	0.3246 (3)	0.0523 (9)	
H26	0.7514	0.5109	0.2712	0.063*	

### Atomic displacement parameters (Å^2^)

**Table:**

	*U*^11^	*U*^22^	*U*^33^	*U*^12^	*U*^13^	*U*^23^
Pd1	0.02505 (11)	0.02529 (12)	0.03109 (13)	−0.00905 (8)	−0.00203 (8)	−0.00290 (8)
Cl1	0.0361 (3)	0.0310 (3)	0.0325 (3)	−0.0106 (3)	−0.0012 (3)	−0.0077 (3)
Cl2	0.0451 (4)	0.0345 (4)	0.0398 (4)	−0.0227 (3)	−0.0061 (3)	0.0001 (3)
Cl3	0.0404 (4)	0.0322 (3)	0.0349 (4)	−0.0187 (3)	−0.0071 (3)	−0.0019 (3)
N1	0.0222 (10)	0.0309 (11)	0.0263 (11)	−0.0128 (9)	0.0024 (8)	−0.0077 (9)
N2	0.0240 (10)	0.0234 (10)	0.0266 (11)	−0.0076 (8)	0.0017 (8)	−0.0045 (8)
C1	0.0226 (11)	0.0292 (12)	0.0228 (12)	−0.0124 (10)	−0.0016 (9)	−0.0058 (10)
C2	0.0307 (13)	0.0370 (14)	0.0221 (12)	−0.0210 (11)	−0.0017 (10)	−0.0016 (10)
C3	0.0442 (16)	0.0407 (16)	0.0283 (14)	−0.0262 (14)	0.0002 (12)	−0.0045 (12)
C4	0.066 (2)	0.0516 (19)	0.0290 (15)	−0.0426 (17)	−0.0017 (14)	−0.0026 (13)
C5	0.061 (2)	0.075 (2)	0.0286 (15)	−0.053 (2)	0.0020 (14)	−0.0060 (15)
C6	0.0392 (16)	0.068 (2)	0.0314 (15)	−0.0353 (16)	0.0045 (12)	−0.0124 (14)
C7	0.0310 (13)	0.0457 (16)	0.0257 (13)	−0.0222 (12)	0.0001 (10)	−0.0100 (12)
C8	0.0266 (13)	0.0446 (16)	0.0312 (14)	−0.0168 (12)	0.0067 (11)	−0.0145 (12)
C9	0.0309 (13)	0.0267 (12)	0.0231 (12)	−0.0122 (11)	−0.0012 (10)	−0.0069 (10)
C10	0.0305 (13)	0.0334 (14)	0.0291 (13)	−0.0158 (11)	−0.0002 (10)	−0.0087 (11)
C11	0.0462 (17)	0.0453 (17)	0.0333 (15)	−0.0279 (14)	0.0031 (13)	−0.0128 (13)
C12	0.069 (2)	0.0345 (15)	0.0322 (15)	−0.0291 (15)	0.0032 (14)	−0.0091 (12)
C13	0.0541 (19)	0.0277 (14)	0.0371 (16)	−0.0095 (13)	−0.0034 (14)	−0.0075 (12)
C14	0.0335 (14)	0.0338 (14)	0.0334 (15)	−0.0079 (12)	−0.0008 (11)	−0.0119 (12)
C15	0.0239 (11)	0.0238 (11)	0.0218 (11)	−0.0102 (9)	0.0020 (9)	−0.0063 (9)
C16	0.0230 (11)	0.0279 (12)	0.0283 (13)	−0.0088 (10)	−0.0011 (10)	−0.0079 (10)
C17	0.0376 (15)	0.0306 (14)	0.0286 (14)	−0.0117 (12)	−0.0057 (11)	−0.0023 (11)
C18	0.0493 (17)	0.0413 (16)	0.0288 (14)	−0.0287 (14)	0.0013 (12)	−0.0017 (12)
C19	0.0328 (14)	0.0503 (18)	0.0358 (15)	−0.0254 (14)	0.0069 (12)	−0.0079 (13)
C20	0.0234 (12)	0.0349 (14)	0.0321 (14)	−0.0103 (11)	0.0003 (10)	−0.0062 (11)
C21	0.0351 (16)	0.0365 (17)	0.079 (3)	−0.0153 (14)	−0.0067 (16)	−0.0175 (17)
C22	0.0331 (15)	0.064 (2)	0.0496 (19)	−0.0235 (15)	0.0041 (14)	−0.0259 (17)
C23	0.0314 (15)	0.0464 (18)	0.056 (2)	−0.0190 (14)	−0.0093 (14)	0.0010 (15)
C24	0.0276 (14)	0.0388 (17)	0.078 (3)	−0.0059 (13)	−0.0101 (15)	−0.0242 (17)
C25	0.0315 (15)	0.089 (3)	0.047 (2)	−0.0249 (18)	0.0012 (14)	−0.031 (2)
C26	0.0394 (17)	0.059 (2)	0.058 (2)	−0.0292 (17)	−0.0142 (16)	0.0092 (17)

### Geometric parameters (Å, °)

**Table:**

Pd1—Cl2	2.2635 (7)	C11—C12	1.386 (5)
Pd1—Cl1	2.2929 (7)	C11—H11	0.9500
Pd1—Cl3^i^	2.3292 (7)	C12—C13	1.374 (5)
Pd1—Cl3	2.3374 (7)	C12—H12	0.9500
Pd1—Pd1^i^	3.4060 (4)	C13—C14	1.381 (4)
Cl3—Pd1^i^	2.3292 (7)	C13—H13	0.9500
N1—C1	1.343 (3)	C14—H14	0.9500
N1—C9	1.426 (3)	C15—C16	1.383 (3)
N1—C8	1.481 (3)	C15—C20	1.388 (3)
N2—C1	1.327 (3)	C16—C17	1.386 (4)
N2—C15	1.424 (3)	C16—H16	0.9500
N2—H2	0.8800	C17—C18	1.380 (4)
C1—C2	1.456 (4)	C17—H17	0.9500
C2—C3	1.388 (4)	C18—C19	1.382 (4)
C2—C7	1.391 (4)	C18—H18	0.9500
C3—C4	1.381 (4)	C19—C20	1.390 (4)
C3—H3	0.9500	C19—H19	0.9500
C4—C5	1.397 (5)	C20—H20	0.9500
C4—H4	0.9500	C21—C22	1.367 (5)
C5—C6	1.381 (5)	C21—C26	1.369 (5)
C5—H5	0.9500	C21—H21	0.9500
C6—C7	1.391 (4)	C22—C23	1.374 (5)
C6—H6	0.9500	C22—H22	0.9500
C7—C8	1.490 (4)	C23—C24	1.377 (5)
C8—H8A	0.9900	C23—H23	0.9500
C8—H8B	0.9900	C24—C25	1.371 (5)
C9—C10	1.385 (4)	C24—H24	0.9500
C9—C14	1.392 (4)	C25—C26	1.393 (5)
C10—C11	1.386 (4)	C25—H25	0.9500
C10—H10	0.9500	C26—H26	0.9500
			
Cl2—Pd1—Cl1	91.32 (3)	C11—C10—H10	120.3
Cl2—Pd1—Cl3^i^	91.00 (3)	C10—C11—C12	119.8 (3)
Cl1—Pd1—Cl3^i^	177.33 (3)	C10—C11—H11	120.1
Cl2—Pd1—Cl3	176.86 (3)	C12—C11—H11	120.1
Cl1—Pd1—Cl3	91.47 (3)	C13—C12—C11	120.9 (3)
Cl3^i^—Pd1—Cl3	86.25 (3)	C13—C12—H12	119.6
Cl2—Pd1—Pd1^i^	134.20 (2)	C11—C12—H12	119.6
Cl1—Pd1—Pd1^i^	134.48 (2)	C12—C13—C14	119.8 (3)
Cl3^i^—Pd1—Pd1^i^	43.218 (17)	C12—C13—H13	120.1
Cl3—Pd1—Pd1^i^	43.030 (18)	C14—C13—H13	120.1
Pd1^i^—Cl3—Pd1	93.75 (3)	C13—C14—C9	119.6 (3)
C1—N1—C9	128.5 (2)	C13—C14—H14	120.2
C1—N1—C8	111.3 (2)	C9—C14—H14	120.2
C9—N1—C8	119.7 (2)	C16—C15—C20	121.3 (2)
C1—N2—C15	127.3 (2)	C16—C15—N2	119.6 (2)
C1—N2—H2	116.3	C20—C15—N2	119.0 (2)
C15—N2—H2	116.4	C15—C16—C17	119.0 (2)
N2—C1—N1	126.8 (2)	C15—C16—H16	120.5
N2—C1—C2	124.2 (2)	C17—C16—H16	120.5
N1—C1—C2	108.9 (2)	C18—C17—C16	120.5 (3)
C3—C2—C7	122.0 (3)	C18—C17—H17	119.8
C3—C2—C1	130.0 (3)	C16—C17—H17	119.8
C7—C2—C1	107.7 (2)	C17—C18—C19	120.1 (3)
C4—C3—C2	117.3 (3)	C17—C18—H18	120.0
C4—C3—H3	121.3	C19—C18—H18	120.0
C2—C3—H3	121.3	C18—C19—C20	120.4 (3)
C3—C4—C5	120.9 (3)	C18—C19—H19	119.8
C3—C4—H4	119.5	C20—C19—H19	119.8
C5—C4—H4	119.5	C15—C20—C19	118.7 (3)
C6—C5—C4	121.7 (3)	C15—C20—H20	120.6
C6—C5—H5	119.2	C19—C20—H20	120.6
C4—C5—H5	119.2	C22—C21—C26	120.6 (3)
C5—C6—C7	117.7 (3)	C22—C21—H21	119.7
C5—C6—H6	121.2	C26—C21—H21	119.7
C7—C6—H6	121.2	C21—C22—C23	120.4 (3)
C2—C7—C6	120.4 (3)	C21—C22—H22	119.8
C2—C7—C8	109.5 (2)	C23—C22—H22	119.8
C6—C7—C8	130.0 (3)	C22—C23—C24	119.6 (3)
N1—C8—C7	102.4 (2)	C22—C23—H23	120.2
N1—C8—H8A	111.3	C24—C23—H23	120.2
C7—C8—H8A	111.3	C25—C24—C23	120.3 (3)
N1—C8—H8B	111.3	C25—C24—H24	119.9
C7—C8—H8B	111.3	C23—C24—H24	119.9
H8A—C8—H8B	109.2	C24—C25—C26	119.9 (3)
C10—C9—C14	120.6 (3)	C24—C25—H25	120.0
C10—C9—N1	120.8 (2)	C26—C25—H25	120.0
C14—C9—N1	118.6 (2)	C21—C26—C25	119.2 (3)
C9—C10—C11	119.3 (3)	C21—C26—H26	120.4
C9—C10—H10	120.3	C25—C26—H26	120.4
			
Cl1—Pd1—Cl3—Pd1^i^	−178.61 (3)	C8—N1—C9—C10	−133.0 (3)
Cl3^i^—Pd1—Cl3—Pd1^i^	0.0	C1—N1—C9—C14	−144.4 (3)
C15—N2—C1—N1	21.0 (4)	C8—N1—C9—C14	44.9 (3)
C15—N2—C1—C2	−155.2 (2)	C14—C9—C10—C11	2.1 (4)
C9—N1—C1—N2	15.2 (4)	N1—C9—C10—C11	−180.0 (2)
C8—N1—C1—N2	−173.5 (2)	C9—C10—C11—C12	−1.2 (4)
C9—N1—C1—C2	−168.2 (2)	C10—C11—C12—C13	−0.9 (5)
C8—N1—C1—C2	3.1 (3)	C11—C12—C13—C14	2.0 (5)
N2—C1—C2—C3	−1.4 (5)	C12—C13—C14—C9	−1.1 (4)
N1—C1—C2—C3	−178.2 (3)	C10—C9—C14—C13	−1.0 (4)
N2—C1—C2—C7	173.0 (2)	N1—C9—C14—C13	−178.9 (2)
N1—C1—C2—C7	−3.8 (3)	C1—N2—C15—C16	42.8 (4)
C7—C2—C3—C4	−1.3 (4)	C1—N2—C15—C20	−139.8 (3)
C1—C2—C3—C4	172.4 (3)	C20—C15—C16—C17	1.7 (4)
C2—C3—C4—C5	0.1 (5)	N2—C15—C16—C17	179.1 (2)
C3—C4—C5—C6	0.9 (5)	C15—C16—C17—C18	−0.4 (4)
C4—C5—C6—C7	−0.7 (5)	C16—C17—C18—C19	−0.7 (5)
C3—C2—C7—C6	1.6 (4)	C17—C18—C19—C20	0.6 (5)
C1—C2—C7—C6	−173.4 (3)	C16—C15—C20—C19	−1.8 (4)
C3—C2—C7—C8	177.9 (3)	N2—C15—C20—C19	−179.2 (2)
C1—C2—C7—C8	3.0 (3)	C18—C19—C20—C15	0.6 (4)
C5—C6—C7—C2	−0.5 (4)	C26—C21—C22—C23	−0.4 (5)
C5—C6—C7—C8	−176.0 (3)	C21—C22—C23—C24	0.1 (5)
C1—N1—C8—C7	−1.3 (3)	C22—C23—C24—C25	0.1 (5)
C9—N1—C8—C7	170.9 (2)	C23—C24—C25—C26	−0.1 (5)
C2—C7—C8—N1	−1.1 (3)	C22—C21—C26—C25	0.3 (5)
C6—C7—C8—N1	174.7 (3)	C24—C25—C26—C21	−0.1 (5)
C1—N1—C9—C10	37.6 (4)		

Symmetry codes: (i) −*x*, −*y*+2, −*z*.

### Hydrogen-bond geometry (Å, °)

**Table:**

*D*—H···*A*	*D*—H	H···*A*	*D*···*A*	*D*—H···*A*
N2—H2···Cl1	0.88	2.37	3.242 (2)	171

## References

[bb1] Altomare, A., Burla, M. C., Camalli, M., Cascarano, G. L., Giacovazzo, C., Guagliardi, A., Moliterni, A. G. G., Polidori, G. & Spagna, R. (1999). *J. Appl. Cryst.* **32**, 115–119.

[bb2] Bartczak, T. J., Michalska, Z. M., Ostaszewski, B., Sobota, P. & Strzelec, K. (2001). *Inorg. Chim. Acta*, **319**, 229–234.

[bb3] Chitanda, J. M., Prokopchuk, D. E., Quail, J. W. & Foley, S. R. (2008). *Organometallics*, **27**, 2337–2345.

[bb4] Fábry, J., Dušek, M., Fejfarová, K., Krupková, R., Vaněk, P. & Němec, I. (2004). *Acta Cryst.* C**60**, m426–m430. 10.1107/S0108270104016725 15345822

[bb5] Farrugia, L. J. (1997). *J. Appl. Cryst.* **30**, 565.

[bb6] Lassahn, P.-G., Lozan, V. & Janiak, C. (2003). *Dalton Trans.* pp. 927–935. 10.1039/b613789j 17160174

[bb7] Nonius (1998). *COLLECT* Nonius BV, Delft,The Netherlands.

[bb8] Ojwach, S. O., Guzei, I. A., Darkwa, J. & Mapolie, S. F. (2007). *Polyhedron*, **26**, 851–861.

[bb9] Otwinowski, Z. & Minor, W. (1997). *Methods in Enzymology*, Vol. 276, *Macromolecular Crystallography*, Part A, edited by C. W. Carter Jr & R. M. Sweet, pp. 307–326. London: Academic Press.

[bb10] Schupp, B. & Keller, H.-L. (2001). *Z. Anorg. Allg. Chem.* **627**, 357–364.

[bb11] Sheldrick, G. M. (2008). *Acta Cryst.* A**64**, 112–122. 10.1107/S0108767307043930 18156677

[bb13] Yang, S.-R., Jiang, H.-F., Li, Y.-Q., Chen, H.-J., Luo, W. & Xu, Y.-B. (2008). *Tetrahedron*, **64**, 2930–2937.

